# Progress in Multiple Sclerosis Genetics

**DOI:** 10.2174/138920212803759695

**Published:** 2012-12

**Authors:** An Goris, Ine Pauwels, Bénédicte Dubois

**Affiliations:** Laboratory for Neuroimmunology, Section of Experimental Neurology, KU Leuven, Leuven, Belgium

**Keywords:** Multiple sclerosis, Genetics, Genome-wide association, Risk, Linkage, Single nucleotide polymorphism.

## Abstract

A genetic component in the susceptibility to multiple sclerosis (MS) has long been known, and the first and major genetic risk factor, the HLA region, was identified in the 1970’s. However, only with the advent of genome-wide association studies in the past five years did the list of risk factors for MS grow from 1 to over 50. In this review, we summarize the search for MS risk genes and the latest results. Comparison with data from other autoimmune and neurological diseases and from animal models indicates parallels and differences between diseases. We discuss how these translate into an improved understanding of disease mechanisms, and address current challenges such as genotype-phenotype correlations, functional mechanisms of risk variants and the missing heritability.

## INTRODUCTION

Multiple sclerosis (MS) is a common neurological disorder characterized by inflammation, demyelination and axonal loss [1]. The disease typically affects young adults and leads to significant physical and cognitive disability. Around 2.5 million people are affected worldwide. Epidemiological studies have clearly demonstrated the involvement of genetic as well as environmental factors. The disease is most common in individuals of Caucasian ancestry, with highest prevalence in Northern Europe [1]. The life-time risk of MS increases with familial history of the disease and ranges from 0.2% in the general European population to 2-4% in siblings of MS patients (sibling recurrence risk 10-20) and 30% in monozygotic twins of MS patients [2-4]. Studies on adoptees, half- and step-siblings have indicated that this increased familial risk is mainly due to shared genetic factors and not to shared lifestyle [5-7]. Nevertheless, there is evidence for the influence of environmental factors such as Epstein Barr virus infection and vitamin D levels on the risk of disease [8]. This review focuses on the recent progress in identifying which genetic factors influence the risk of MS, how these findings shed light on the pathogenesis of the disease and what their translational potential is.

## MULTIPLE SCLEROSIS GENETICS

### The HLA Region

The Human Leukocyte Antigen (HLA) region on chromosome 6p21 is characterized by an exceptional degree of polymorphism or genetic variation between individuals in the population. With the use of serology, these were amongst the first polymorphisms that could be studied in the ‘70s. It became quickly evident that the HLA region plays a role in nearly all immune-related disorders, including MS [9, 10]. Strongest association was observed with the DR2 serotype [11], which was refined with DNA-based typing methods to the DRB1*1501 allele [12-14]. The DRB1*1501 allele frequency is between 3 and 20% in the European population, with population frequency increasing with population risk of MS from Southern to Northern Europe [15]. Each copy of this allele increases the risk of MS approximately 3-fold, making it the strongest genetic risk factor for MS [16]. Many studies have suggested that additional risk factors within the HLA region exist, but unraveling these has been hampered by the exceptional nature of the HLA region: the vast degree of polymorphism, the extensive linkage disequilibrium over long distances and the high gene density. In the most recent international genome-wide association study, five alleles at three different loci in the HLA region influence MS susceptibility: the HLA-DRB1*1501, *0301 and *1303 alleles, the HLA-A*0201 allele, and a variant likely reflecting the HLA-DPB1*0301 allele [17]. These alleles correspond to changes in risk between 26% and 200%. Together, the HLA alleles explain at most 20% of the sibling recurrence risk for MS [16].

### Early Days: Linkage Studies in Multiplex Families

The linkage strategy, looking for cosegregation of a genomic region with a disease within multiplex families, has proven extremely effective in mapping monogenic forms of neurological diseases. In MS, 20% of patients report a relative with the disease, but families with more than four affected relatives over more than one generation are extremely rare [18]. This limits the use of linkage studies in large families in MS. Early linkage studies have instead attempted to combine linkage signals over many smaller families (mainly affected sibling pairs) employing microsatellite maps. However, these studies suffered from a lack of power and could not even distinguish the role of the HLA region beyond doubt at genome-wide significance levels [19-21]. Nevertheless, a significant excess of genetic sharing between affected relatives over what is expected was observed. This confirms the role of genetic susceptibility in the development of the disease but illustrates that studies were not powerful enough to identify individual risk factors. A definitive linkage study using a high-density single nucleotide polymorphism (SNP) map in 730 multiplex families resulted in overwhelming evidence for the HLA region (LOD score 11.66). However, no other regions reached genome-wide significant LOD scores of 3, and there was a vast gap between the score for the HLA region and the next LOD scores (≤2.45) reflecting suggestive evidence only [22]. This information was instrumental in estimating the effect size of genetic risk factors outside of the HLA region that can realistically be anticipated and the corresponding choice of strategies to identify them [23]. 

### The Era of Genome-Wide Association Studies: The ‘Common Disease - Common Variant’ Hypothesis

With decreasing effect size of a risk factor, the number of affected sibling pairs required for linkage studies increases dramatically. If we assume that common variants each with a modest effect on disease risk contribute substantially to common diseases such as MS (‘common disease - common variant’ hypothesis), association studies in large study populations are much more suited than linkage studies [24]. Early candidate-gene association studies in a few hundred of cases and controls led to contradicting results and frustratingly little progress. Association studies became, however, possible on a large scale around five years ago thanks to the knowledge provided by the Human Genome Sequence and the Human HapMap project [25], the technological advances with the development of micro-arrays, and the collection of large study populations through international collaboration. Association studies compare a group of affected individuals (cases) with a group of controls and look for differences in the frequency of a variant between both groups. Because of the large number (typically 500,000 to 1,000,000) of tests required to cover most (>80%) of the common variation throughout the genome in genome-wide association studies (GWAS), stringent significance thresholds are needed. It has been demonstrated both theoretically and empirically that P-values of 10^-7^ to 5x10^-8^ are highly indicative of genuine associations that replicate in independent follow-up studies [23, 26].

Thirty-five years after the first report on the role of the HLA region in MS, association studies identified the first genetic risk factors for MS outside of the HLA region: variants in the cytokine receptor genes interleukin-2 receptor alpha (*IL2RA*) and interleukin-7 receptor (*IL7R*) [27-29]. In the following years, genome-wide and candidate gene association studies increased the number of established or suggestive risk variants to 26 [30-44]. Whereas the first GWAS typically included up to 3000 cases, this number was more than tripled in the most recent genome-wide association study performed by a large international consortium and comprising 9,772 cases and 17,376 controls from 15 study populations of European ancestry [16]. This increased sample size and power is reflected in the number of risk variants detected: the study confirmed 23 out of 26 previously reported risk loci and identified another 34, bringing the total number of MS risk variants outside of the HLA region to 57 [16] (Table **1**). Since, additional GWAS published keep the list of risk variants growing [45]. Established risk variants are common in the general population, with frequencies of the risk alleles between 13% and 92% [16]. Such high frequencies, with risk alleles in many instances being more common than the corresponding protective alleles, are at first sight counter-intuitive. This underlines, however, once again the multifactorial nature of most autoimmune diseases as well as the pleiotropic effects of immune-related genes. Different functions of these genes may contribute to adaptation and selection of specific variants [46, 47], or environmental changes in modern societies may expose the disease risk associated with the variants [48]. Each risk allele increases the risk of MS 1.08 to 1.22-fold [16]. These modest effect sizes are the reason why large study populations were needed to identify them, as compared to only around 100 cases and controls needed to detect the major signal from the HLA region with an odds ratio of 3 [23]. The established 57 variants together with the HLA variants are estimated to explain 25% of the sibling recurrence risk [16].

Many more common risk variants are hidden below the stringent genome-wide significance threshold in current GWAS. Indeed, studies looking at the collective effect of many variants together (polygenic or “en masse” models) demonstrate that the current set of risk alleles is just the tip of the iceberg likely to include hundreds of variants with modest effects and thousands of variants with very small effects [49]. Studies in even larger study populations are under way and are expected to identify part of the remaining variants with modest effect size.

GWAS are most powerful in identifying regions of association. These regions are typically 384 kb in size and contain an average of 6 genes (range 0-33) [16]. However, GWAS meet their limitations in identifying which are the most likely functional genes and variants within these regions. Such fine-mapping involves detailed follow-up genotyping as well as functional work (see below). Detailed follow-up studies of risk genes for MS using an “Immunochip”, a micro-array containing dense SNP maps of known risk genes for autoimmune diseases, are currently ongoing [50].

### Next-Generation Sequencing: Less Common and Rare Variants

GWAS are based on the ‘common disease - common variant’ hypothesis, and effectively screen the vast majority (>80%) of the 10 to 15 million common (>5% minor allele frequency in the general population) variants in the genome [51]. The effects of the billions of less common (1-5% minor allele frequency) or rare (<1% minor allele frequency) variants on the risk of MS go undetected with current micro-arrays. Linkage studies in affected sibling pairs provide upper estimates for the effect size that can be expected from such less common or rare variants [23, 52]. A rare variant with a frequency of 0.2% and an odds ratio of 20 would result in >60% allele sharing in affected siblings and should have been detected in linkage studies employing a few hundred affected sibling pairs [24]. Pilot studies in other autoimmune diseases indeed suggest that there are fewer rare variants with large effect sizes (odds ratios >3) in autoimmune diseases than in other multifactorial disorders [53, 54]. However, less common or rare variants with weak to moderate effects may contribute to MS susceptibility. Indeed, the first few examples are emerging.

A common variant in the *TNFRSF1A* gene has recently been identified as a risk variant for MS [16, 39, 43]. Rare and less common variants in the same gene cause Tumor Necrosis Factor Receptor-Associated Periodic Syndrome (TRAPS, MIM 142680). Symptoms of this disease were reported at increased frequency amongst MS patients [55]. One of the mildest TRAPS variants, with an allele frequency of 1% in the general population, was subsequently observed to be overrepresented amongst MS patients (allele frequency 3%), corresponding to a doubling in the risk of MS, larger than the effect of most common variants [39, 56]. Two studies suggest that the rare and common variant act independently [39, 56], but this remains to be investigated further.

As a strategy to identify rare variants, a recent study applied exome sequencing in index patients from 43 multiplex families with at least 4 affected individuals. No rare functional variant was present in more than one family. Subsequently, the authors looked for rare variants in genes already implicated by common MS risk variants and identified three such variants, including one in *CYP27B1*. After follow-up in a total of 3564 MS patients and 1873 controls, they suggest association of five rare variants in this gene with MS [57]. In an autosomal recessive manner, these variants cause vitamin D dependent rickets type 1 (VDDR1, OMIM: 264700), a childhood onset disease caused by the lack of 1-alpha-hydroxylase enzyme converting 25-hydroxyvitamin D to its biologically active form 1,25-hydroxyvitamin D or calcitriol. In this study, carriers of any of these five rare variants appear to be at increased risk of developing MS (odds ratio = 4.7) [57].

### The Immunogenetics of Multiple Sclerosis

With an established list of 5 HLA and 57 non-HLA risk factors for MS, we can finally start to consider the picture of pathways emerging and convert lists of variants into an improved understanding of disease mechanisms.

A hypothesis-free investigation of regions implicated by GWAS indicates that these regions are heavily enriched for immunological genes (P=10^-8^). Considering the genes nearest to each of the association signals, 30% of these *versus* 7% of all human genes are annotated in public databases as having an immunological function [16]. The role of the immune system is further supported by the overlap with other diseases. Nearly half of MS risk genes are shared with other autoimmune diseases, most importantly celiac disease, Crohn’s disease, primary biliary cirrhosis, type 1 diabetes, and rheumatoid arthritis [16, 58] (Table **1**). Often, the same variants are associated in the same direction with several autoimmune diseases and hence appear to influence susceptibility to autoimmunity in general. An example is the B cell specific transcription factor *BACH2* in which variants are associated with at least five other autoimmune diseases besides MS: type 1 diabetes [59], Crohn’s disease [60], celiac disease [61], autoimmune thyroiditis [62] as well as vitiligo [63]. Other variants act as risk factors for one whilst being protective against another autoimmune disease: the alleles that increase CD40 expression increase the risk of rheumatoid arthritis and Graves’ disease [64, 65] but protect against MS [16, 36]. A careful examination of the overlaps and differences between diseases can inform us about common and disease-specific pathways [58].

Common MS risk variants do not only tend to cluster within the same genes with common risk variants for other autoimmune diseases. In several instances, common risk variants implicate genes in which rare mutations are known to cause monogenic immune-related diseases characterized by autoimmunity, autoinflammation or immunodeficiency (Table **2**). This may indicate that these key immune system controlling genes, and not other genes within the same region of association, are functional in susceptibility to MS.

Table **3** provides an overview of the effect of genes in mice, as well as in the mouse model for MS (experimental autoimmune encephalomyelitis - EAE) for the most likely functional genes near each of the established MS risk variants. This points once again to different particular pathways of the immune system implied by currently known genetic risk factors for MS. 

The current state of knowledge of MS genetics hence provides us with new insights into the pathogenesis of the disease and implicates a major role for the immune system, and specifically for lymphocyte differentiation and proliferation. Selected candidate genes within the regions of association implicated by GWAS include cytokines (e.g. *IL12A, IL12B*) and cytokine receptors (e.g. *IL7R, IL2RA, IL22RA*), co-stimulatory molecules (e.g. *CD58, CD6, CD40, CD80, CD86*), and signal transducer molecules (e.g. *TYK2, STAT3*) [16].

### The Relative Absence of the Neurological Component Amongst Common MS Risk Variants

The neurodegenerative component of MS that is apparent in pathological studies is less well reflected in the list of currently known genetic risk factors. None of the risk genes overlap with those for neurodegenerative diseases such as amyotrophic lateral sclerosis, Alzheimer’s disease or Parkinson’s disease. Only a few candidate genes implied by genome-wide association studies have an obvious neurological function. Mutations in β-A-mannosidase (*MANBA)* (OMIM: 248510) and galactosylceramidase (*GALC)* (OMIM: 245200) lead to lysosomal storage disorders with neurological symptoms or myelination problems, and anti-galactosyl ceramide antibodies are specific for MS [66] (Table **2**). Two members of the kinesin family have been implied in the risk of MS. The first, *KIF1B*, could not be replicated in independent study populations [67, 68]. Variation in the second, *KIF21B*, has an effect in the same direction on the risk of MS as well as other autoimmune diseases such as ankylosing spondylitis [69] and inflammatory bowel disease [60], implying that its immunological instead of neurological function may be key to explaining the association [32, 35]. 

Common MS risk variants implicated by GWAS are enriched in the regulatory DNA sequences of immune-related cell types but moderately depleted in the regulatory DNA sequences of brain tissue, suggesting a less important role of neural than immune-related gene expression regulatory elements in the pathogenesis of MS [70].

It remains possible that some of the yet unknown risk variants, especially less common and rare ones, will provide a genetic basis for the neurodegenerative component in MS. Indeed, common variants appear to play a more substantial role in autoimmune compared to other complex diseases [71]. This may reflect a history of selection and adaptation for variation in regions controlling the immune system [72, 73]. In neurodegenerative diseases, on the other hand, an increasing number of apparently sporadic cases turn out to harbor rare risk variants [74].

### Disease Heterogeneity Left Unexplained

Clinicians observe MS as a remarkably heterogeneous disease, in terms of age at onset, disease course (bout onset or primary progressive) and disease activity (relapse rate and accumulation of disability). Studies indicate that there may be a genetic component in some of these clinical characteristics [75]. In contrast to the susceptibility of disease where substantial progress is being made, our understanding of the factors underlying this clinical heterogeneity is very limited. In the most recent and largest GWAS, no genetic factors contributing to differences in disease course (bout onset versus primary progressive) or severity (measured by the Multiple Sclerosis Severity Scale [76]) were observed [16]. The strongest genetic risk factor, the HLA region, is indeed shared between bout onset and primary progressive MS [77] and the genetic load based on all currently known genetic risk factors is increased in both bout onset and primary progressive MS patients compared to healthy controls [78] (Goris A., unpublished data). 

There was limited evidence only for factors contributing to differences in age at onset: a higher genetic risk tends to be correlated with an earlier age at onset [16]. For example, each copy of the HLA-DRB1*15:01 allele a patient carries decreases age at onset by an average of 10.6 months [16], in line with previous reports [77, 79-82].

Clinical heterogeneity may be an endpoint reflecting many different disease processes. Using intermediate measures (endophenotypes) that capture specific biological processes, are at least in part genetically determined and can be measured accurately may hence be more useful. Examples of such endophenotypes are antibody production in the cerebrospinal fluid and magnetic resonance imaging (MRI) based measurements. Relatively small studies of the HLA region have so far suggested that different HLA alleles are associated with oligoclonal band positive or negative MS [83-85]. In one of the first GWAS for an imaging-based trait, association with glutamate concentrations as a proxy for neurodegenerative processes is reported [86]. More studies investigating on a systematic basis such endophenotypes reflecting specific disease processes are awaited.

Another aspect of heterogeneity important to clinical practice is that of response to treatment. First examples of investigations of this trait are discussed below.

### Missing or Hidden Heritability

As described above, currently known risk variants for MS are estimated to account for approximately 25% of the sibling recurrence risk [16], with an additional fraction explained by the collective effect of hundreds to thousands of common variants with modest to small effects [49, 70]. However, a substantial proportion of the genetic risk of MS remains unexplained [71]. 

A first possible explanation for this missing or hidden heritability implicates novel sources of genetic risk such as less common and rare variants (see previously), non-SNP risk variants such as structural variants (copy number variants and inversions) and inherited epigenetic variation. It has not been possible to explore these sources of genetic variation systematically and in sufficiently large sample sizes as yet. 

A second explanation for the missing or hidden heritability is an underestimation of the fraction of the genetic risk that is already explained by currently known variants. First, there are indications that current estimates of familial clustering and heritability may be overestimated, and hence the proportion currently explained underestimated [87, 88]. Second, GWAS are most powerful in detecting regions of association but their inherent limitations diminish their ability to correctly estimate the effect size. Markers present on the micro-arrays used in GWAS may capture the true functional variants imperfectly, and hence dilute the association. Evidence also suggests that the same genes tend to harbor several variants independently associated with MS [16]. Hence, a risk and protective variant that are present together on the same haplotype may dilute the marginal effect observed. Thirdly, gene-gene and gene-environmental interactions may lead to an underestimation when the effect size is based on single gene associations. 

### From Variant to Function

As in most other complex diseases, the vast majority of risk variants implicated by GWAS are not located in the coding region or correlated with coding variants, but are situated in introns, promoters or intergenic regions [47]. Such variants are highly enriched in regulatory DNA sites, indicating that they may modulate local chromatin accessibility [70]. The identification of target genes of regulatory GWAS variants is complicated because they can be located at great distances from the gene(s) they control and function through long-range regulatory interactions [70].

A role for some genes is suggested by data on the effect of knocking out the homologous gene in mice (Table **3**). For example, tyrosine kinase *(Tyk2) *knockout (KO) mice showed complete resistance against EAE with no infiltration of CD4^+^ T cells in the spinal cord and reduced Th1 cells in the periphery. Furthermore, induction of EAE in MOG-primed *Tyk2* KO mice by transferring wildtype (WT) Th1 cells suggests that the diminished Th1 response is involved in the mechanism for disease resistance [89]. This corroborates data in humans where the MS risk variant influences the Th1/Th2 balance [90] (Table **4**). However, extrapolation between the mouse model and human disease and between gene knock-out and more subtle polymorphism is not always possible. The MS susceptibility gene *CD58* for example does not have an orthologue in mice. The tumor necrosis factor receptor superfamily member 1A *(Tnfrsf1a)* KO mouse is resistant to EAE and administration of neutralizing antibodies delayed the onset of disease and clinical symptoms in this animal model [91-93]. These findings led to a clinical trial of anti-tumor necrosis factor (anti-TNF) antibodies as a therapy for MS patients, which unexpectedly caused worsening of the patients’ condition [94, 95].

Given the limitations of animal models described above, human material is essential to examine the mechanism of action of genetic risk variants directly in humans. Many studies have demonstrated the appropriateness of the peripheral blood system to capture variation that is of importance for MS [96]. Possible mechanisms of action include effects on splicing or on gene expression. Examples of both mechanisms are listed in Table **4**. The MS risk allele in *TNFRSF1A* increases splicing of exon 6 encoding the transmembrane domain and hence directs the production of a natural antagonist of TNF-α, which mirrors the outcome of the clinical trials described above [97]. An elegant pioneering study investigated the effect of risk variants in *IL2RA* on the immunological phenotype. The authors demonstrate that variants involved in susceptibility to MS are correlated with IL2RA (CD25) expression on naive T cells and monocytes, whereas variants that are neutral in MS but associated with type 1 diabetes are correlated with changes in expression on memory T cells [98]. Studies on the functional effects of SNPs will be helpful in demonstrating pathways underlying MS and overlaps and differences with other autoimmune diseases.

### Translational Potential

The potential for currently known risk variants as a prediction tool for MS is limited, as in other complex diseases, and is not the aim of genetic studies, as has extensively been discussed elsewhere [71, 88]. More important is the hope that improved understanding of the disease pathogenesis will translate to improved treatment of patients, either through the identification of novel targets for treatment or by optimizing treatment choice (personalized medicine). As a proof-of-principle, it is notable that two of the targets of monoclonal antibodies currently being used or investigated for treatment of MS (*VCAM1* - natalizumab, *IL2RA* - daclizumab) are implicated by GWAS [16]. A few GWAS have been performed to search for determinants of response to the first-line interferon-beta therapy in MS, but results await further validation [99]. A recent study investigated the occurrence of major side effects upon treatment with alemtuzumab. Genetically determined interleukin-21 (IL-21) levels appear to predict partly who is at risk of secondary autoimmunity, an important side effect in a subset of treated patients [100]. An example where GWAS mirrors and informs clinical experience is that of *TNFRSF1A*, the receptor for TNF-α. Variants in this gene are associated with susceptibility to MS and primary biliary cirrhosis but not with other autoimmune diseases [16, 39, 101]. The MS risk allele in this gene directs expression of a soluble form of TNFR1 that acts as a natural TNF antagonist [97]. This mirrors the outcome of clinical trials where TNF antagonists worsened the disease [94, 95] and the experience with the use of TNF antagonists in non-MS autoimmune diseases such as Crohn’s disease and rheumatoid arthritis where side effects include clinical onset of MS and isolated demyelinating diseases [102]. It remains to be investigated whether stratification by the genetic variant can identify a subset of individuals prone to these effects.

## CONCLUSION

The field of MS genetics has come a long way, with the list of genetic risk factors having doubled over the past year to >50. On the basis of this list a picture is emerging of key immunological genes and pathways being involved in MS susceptibility, with much less representation of common variants in genes with neurological function so far. Current challenges are understanding the functional mechanisms, with a few pioneering studies leading the way. Established risk variants explain only part of the genetic component of MS and the search for the missing heritability goes on with novel tools addressing additional sources of variation that were beyond the reach of systematic investigation so far. The heterogeneity in clinical aspects such as age at onset, disease course and severity remains largely unexplained at the genetic level, with the exception of an effect on age at onset. However, an important aim of genetic studies is translation to novel targets for treatment or optimal treatment choice based on the genetic profile and ‘proof of principle’ examples are hopeful.

## Figures and Tables

**Table 1.** MS Risk Variants Identified in the Most Recent Genome-Wide Association Study (Updated From [16])

Chr	Rs id	Position	Candidate Gene	Risk Allele	Odds Ratio	Risk Allele Freq Ctrls	Tags Autoimmune Disease SNP a
1	rs4648356	2699024	*MMEL1 (TNFRSF14)*	C	1.14	0.68	RA, CeD, UC
1	rs11810217	92920965	*EVI5*	A	1.15	0.25	
1	rs11581062	101180107	*VCAM1*	G	1.12	0.28	
1	rs1335532	116902480	*CD58*	A	1.22	0.87	
1	rs1323292	190807644	*RGS1*	A	1.12	0.82	CeD
1	rs7522462	199148218	*C1orf106 (KIF21B)*	G	1.11	0.73	UC, CeD, CrD, AS
2	rs12466022	43212565	*No gene*	C	1.11	0.73	
2	rs7595037	68500599	*PLEK*	A	1.11	0.56	CeD
2	rs17174870	112381672	*MERTK*	G	1.11	0.76	
2	rs10201872	230814968	*SP140*	A	1.14	0.16	CrD
3	rs11129295	27763784	*EOMES*	A	1.11	0.37	
3	rs669607	28046448	*No gene*	C	1.13	0.48	
3	rs2028597	107041527	*CBLB*	G	1.13	0.92	
3	rs2293370	120702624	*TMEM39A (CD80)*	G	1.13	0.82	
3	rs9282641	123279458	*CD86*	G	1.21	0.90	
3	rs2243123	161192345	*IL12A*	G	1.08	0.27	CeD, PBC
4	rs228614	103797685	*NFKB1 (MANBA)*	G	1.09	0.52	PBC
5	rs6897932	35910332	*IL7R*	G	1.11	0.73	T1D, PBC, UC
5	rs4613763	40428485	*PTGER4*	G	1.2	0.13	CrD, UC, AS
5	rs2546890	158692478	*IL12B*	A	1.11	0.52	Ps, CrD
6	rs12212193	91053490	*BACH2*	G	1.09	0.44	CeD, T1D, CrD, AITD, VIT
6	rs802734	128320491	*THEMIS*	A	1.1	0.70	CeD
6	rs11154801	135781048	*MYB (AHI1)*	A	1.13	0.36	
6	rs17066096	137494601	*IL22RA2*	G	1.14	0.24	
6	rs13192841	138008907	*No gene*	A	1.1	0.27	RA
6	rs1738074	159385965	*TAGAP*	G	1.13	0.58	CeD, CrD
7	rs354033	148920397	*ZNF746*	G	1.11	0.74	
8	rs1520333	79563593	*IL7*	G	1.1	0.26	
8	rs4410871	128884211	*MYC*	G	1.11	0.72	
8	rs2019960	129261453	*PVT1*	G	1.12	0.21	
10	rs3118470	6141719	*IL2RA*	G	1.12	0.33	RA, VIT
10	rs1250550	80730323	*ZMIZ1*	A	1.1	0.34	CeD, IBD
10	rs7923837	94471897	*HHEX*	G	1.1	0.62	
11	rs650258	60588858	*CD6*	G	1.12	0.64	
11	rs630923	118259563	*CXCR5*	C	1.12	0.84	PBC
12	rs1800693	6310270	*TNFRSF1A*	G	1.12	0.41	PBC
12	rs10466829	9767358	*CLECL1*	A	1.09	0.51	T1D
12	rs12368653	56419523	*CYP27B1*	A	1.1	0.48	RA
12	rs949143	122161116	*ARL6IP4*	G	1.08	0.30	
14	rs4902647	68323944	*ZFP36L1*	G	1.11	0.53	CeD, T1D, CrD
14	rs2300603	75075310	*BATF*	A	1.11	0.75	
14	rs2119704	87557442	*GALC (GPR65)*	C	1.22	0.92	
16	rs2744148	1013553	*SOX8*	G	1.12	0.17	
16	rs7200786	11085302	*CLEC16A (CIITA)*	A	1.15	0.46	T1D, PBC
16	rs13333054	84568534	*IRF8*	A	1.11	0.22	RA
17	rs9891119	37761506	*STAT3*	C	1.11	0.35	CrD
17	rs180515	55379057	*RPS6KB1*	G	1.09	0.34	
18	rs7238078	54535172	*MALT1*	A	1.12	0.76	
19	rs1077667	6619972	*TNFSF14*	G	1.16	0.79	
19	rs8112449	10381064	*TYK2 (ICAM3)*	G	1.08	0.67	T1D, Ps
19	rs874628	18165700	*MPV17L2 (IL12RB1)*	A	1.11	0.72	
19	rs2303759	54560863	*DKKL1 (CD37)*	C	1.11	0.26	
20	rs2425752	44135527	*CD40*	A	1.11	0.27	RA
20	rs2248359	52224925	*CYP24A1*	G	1.12	0.60	
20	rs6062314	61880157	*TNFRSF6B*	A	1.16	0.92	CrD, UC
22	rs2283792	20461125	*MAPK1*	C	1.1	0.51	
22	rs140522	49318132	*SCO2*	A	1.1	0.33	

a Tags (r^2^>0.1) a SNP associated with another autoimmune disease (Catalog of Published Genome-wide Association Studies, version 13/07/2012).

a Abbreviations: AITD = autoimmune thyroid disease, AS = ankylosing spondylitis, CrD = Crohn’s disease, CeD = Celiac Disease, IBD = inflammatory bowel disease, PBC = primary biliary cirrhosis, Ps = psoriasis, RA = rheumatoid arthritis, T1D = type 1 diabetes, UC = Ulcerative colitis, VIT = vitiligo

**Table 2.** Overlap of MS Risk Genes Implicated by Genome-Wide Association Studies with Genes Underlying Monogenic Disorders

Gene Implicated by GWAS	Common MS Risk Variants	Monogenic Immune-Related Disease Variants (OMIM) a	Rare MS Risk Variants
**Immunological Diseases**			
*CYP27B1*	rs703842 (upstream) [36]	Vitamin D dependent rickets (AR, OMIM: 264700): many variants including R389H, E189G, L343F, Y413C and R252C	R389H, E189G, L343F, Y413C and R252C (OR=4.7) [57]
*TNFRSF1A*	rs1800693 (intronic) [39]	Tumor necrosis factor receptor-associated periodic syndrome (AD, OMIM: 142680): many variants including R92Q	R92Q (OR=2.2) [39, 56]
*TYK2*	rs34536443 (exonic, P1104A) [37]	Hyper-IgE recurrent infection syndrome (HIES) with atypical mycobacteriosis (AR, OMIM: 611521): 4bp frameshift deletion	Not known
*IRF8*	rs17445836 (downstream) [39]	Severe immunodeficiency with lack of monocytes and dendritic cells (AR): T80A, K108E [103]	Not known
*IL7R*	rs6897932 (exonic, T244I) [27]	Severe combined immunodeficiency (AR, OMIM: 608971): several mutations	Not known
*IL2RA*	rs2104286 (intronic) [28]	Immune deficiency (AR, OMIM: 606367): 4bp frameshift deletion	Not known
*MERTK*	rs17174870 (intronic) [16]	Autosomal retinitis pigmentosa (AR, OMIM: 613862): several mutations	Not known
**Neurological Diseases**			
*MANBA*	rs228614 (intronic) [16]	β- mannosidase deficiency (AR, OMIM: 248510): several mutations	Not known
*GALC*	rs2119704 (downstream) [16]	Krabbe disease (AR, OMIM: 245200): several mutations	Not known

a AR= autosomal recessive, AD = autosomal dominant.

**Table 3.** Established Risk Genes for MS and Their Mouse Models

Gene	Animal Model	Phenotype	EAE	References
*AHI1*	Neuronal cell conditional *Ahi1* KO mice	Reduced signaling through TrkB-BDNF pathway		[104]
*BACH2*	*Bach2*(-/-) mice	Reduction of mature B-cells, required for T-cell independent and dependent IgG response and somatic hypermutation of Ig genes.		[105, 106]
*BATF*	*Batf*(-/-) mice	Impaired Ig secreting B-cell maturation, defect in Th17 differentiation and follicular Th cells.	Resistant to EAE.	[107, 108]
*CBLB*	*Cblb*(-/-) mice	T-cells do not require CD28 for IL-2 production.	More susceptible to EAE after immunization.	[109, 110]
*CD37*	*Cd37*(-/-) mice	Reduced level of IgG1 in the sera of non-immunized mice and alteration of response to T-cell-dependent antigens.		[111]
*CD40*	*Cd40*(-/-) mice	Defective germinal center formation and no IgG, IgA or IgE response to thymus-independent Ag.		[112, 113]
*CD80*	Selective silencing of full-length *Cd80* protein	Role in response of recently activated cells and in sustaining T cell responses.		[114]
*CD86*	*Cd86*(-/-) mice	Fail to switch Ab isotypes and to form germinal centers, altered Th1 and Th2 dependent responses.		[115]
*CXCR5*	*Cxcr5* KO = *Blr1*(-/-) mice	Activated B-cells fail to migrate from T-cell rich to B-cell follicles of the spleen and no development of functional germinal centers in spleen.		[116]
*CYP24A1*	*Cyp24a1*(-/-) mice	Bone malformations during development and defective fracture repair.		[117, 118]
*CYP27B1*	*Cyp27b1*(-/-) mice	Enlarged lymph nodes in the vicinity of the thyroid gland and a reduction of the CD4+ and CD8+ peripheral T lymphocytes.		[119]
*DKKL1*	*Dkkl1*(-/-) mice	Increased testosterone production in testis, secondary to increased expression of *CYP11A* and *CYP17* in the Leydig cells.		[120]
	Conditional *Dkkl1* KO mice (ES, blastocyts)	Is not essential for development or fertility, no observable phenotype.		[121]
*EOMES*	*Eomes*(-/-) mice	Retinal ganglion cells: reduced numbers of nerves, abnormal axonal growth, aberrant myelin sheath.		[122]
	*Tbet* */ *Eomes** ** DKO mice	CD8+ T-cells fail to differentiate into functional killers but develop in IL-17 secreting lineage.		[123, 124]
*GALC*	*Galc*^twi/twi^ mice (spontaneous mouse model, twi=Galc)	Progressive demyelination and accumulation of macrophages in the nervous system.		[125, 126]
	*Galc* transgenic mice	Progressive demyelination and accumulation of macrophages in the nervous system.		[127]
*GPR65*	Transposon-induced *Tdag8*Tp/Tp mice	Attenuation of inhibition of extracellular acidification-induced proinflammatory cytokine production in macrophages.		[128]
	*Tdag8*(-/-) mice	Undetectable pH-dependent cAMP production in thymocytes and splenocytes.		[129]
*HHEX*	*Hex*(-/-) *Rag1*( -/-) mice	Failed Ab response to T-cell independent Ag, reduced number of mature B-cells, pre B-cells, CD5+ B-cells and strongly increased B220-CD19+ B-cells in bone marrow.		[130, 131]
*HLA*	Humanized HLA-DR1/CD4 mice	T cell responses to human MBP 139-154 peptide but no HLA transgene-dependent autoimmune disease was seen.	No spontaneous EAE or EAE after immunization.	[132]
	Humanized HLA-DR2/TCR/CD4 mice		Spontaneous EAE in 4%, after MBP immunization: 90%. When crossed with RAG2-null background 100% spontaneous EAE.	[133]
	Humanized HLA-DR15/TCR mice		Spontaneous EAE in 60%, when crossed with RAG2-null background 100% spontaneous EAE.	[134]
	Humanized HLA-A* 0301/TCR mice	HLA-A3-restricted CD8+ T-cells influence induction of disease and CD4+ T-cells progression.	Spontaneous mild EAE in 4%, after PLP immunization 71% mild EAE.	[135]
	Humanized HLA-A* 0301/HLA-A* 0201/TCR mice	HLA-A2 is protective by inducing deletion of autoreactive clones with high levels of TCR.	No spontaneous EAE or EAE after immunization.	[135]
*IL12A*	*Il12a p35*(-/-) mice	Shift to Th2 response, CD4+ Tcells produce more IL-4 and less IFN-γ.		[136, 137]
*IL12B*	*Il12b p40*(-/-) mice	Impaired IFN-γ production to several stimuli and deficiency in the ability to generate normal Th1 responses.		[138]
	*Il12b p40* knock-in mice	Dendrocytes expressing p40 migrate to draining lymph nodes and promote Th1 differentiation.		[139]
*IL12RB1*	*Il12rb1*(-/-) mice	Only expression of low affinity IL-12-binding sites, splenocytes fail to proliferate or produce IFN-γ in response to IL-12 stimulation.		[140]
*IL2RA*	*Il2ra*(-/-) mice	Enlarged lymphoid tissue due to lymphocyte expansion, reduced proliferative response of T-cells and elevated serum Ig (spontaneous inflammatory bowel disease and anemia).		[141]
*IL7*	ENU*Il7* mice	Drastic reduction in the number of T and B-cell lineages in the peripheral blood and lymphoid organs.		[142]
	*Il7*(-/-) mice	Drastic reduction of lymphoid cells in the thymus, bone marrow and spleen.		[143, 144]
*IL7R*	*Il7r*(-/-) mice	Reduced thymic and peripheral lymphoid cellularity. Absence of γδ T-cell and reduced &amp;alpha;&amp;beta; T-cells and B cells.		[145, 146]
*IRF8*	*Irf8*(-/-) mice	Enhanced proliferation of myeloid, monocytic and lymphoid lineages (similar to human chronic myelogenous leukemia).		[147]
	B-cell specific* Irf8*conditional knockout mice	Enlarged marginal zone and increased numbers of marginal zone and follicular B-cells.		[148]
*MALT1*	*Malt*(-/-) mice	B-cells and T-cells showed decreased activation, proliferation, and IL-2 production in response to TCR ligation.		[149-151]
*MANBA*	*Manba*(-/-) mice	Cytoplasmic vacuolation in central nervous system and visceral organs (resembles human lysosomal storage disease).		[152]
*MAPK1*	Conditional *Erk2* T-cell knockout mice	Decreased thymic cellularity, reduced CD4+ and CD8+ SP thymocytes and DP thymocytes.		[153]
	*Erk2*knockdown mice	Deficit in long term memory in classical fear conditioning.		[154]
	Conditional *Erk2*neuronal cell knockout mice	Important role in cellular proliferation and differentiation during neuronal development as well as in cognition and memory formation.		[155, 156]
*MERTK*	*Mer*^kd mice^ (=knockdown)	Increased TNF-α production upon LPS stimulation.		[157]
*MMEL1*	*Mmel1* KO =* Nep2*(-/-) mice	Higher amyloid-beta.		[158]
*MYB*	T-cell specific* Myb *knockout mice	Reduced SP thymocytes, reduced survival and proliferation of DP thymocytes and reduced proliferation of mature T-cells.		[159, 160]
	Knockdown* Myb* mice	Reduced SP thymocytes.		[161]
*MYC*	*Myc* knock-in mice	Higher proliferation of B-cells in response to activation signals (Burkitt-like B-cell lymphoma).		[162, 163]
	*Myc*(-/-) mice	Not needed for cellular growth but decreased percentage of activated T-cells re-enter the cell cycle.		[164]
*NFKB1*	*Nfkb1*SSAA/SSAA &amp;nbsp;mice (IKK-target serine residues of p105 substituted by alanine)	Less CD4+ regulatory and memory T-cells and blocked ability to provide help to wild type B-cells during antibody response.		[165]
	*NFkb1* *p105*(-/-) mice	Lymphocytic infiltration in lungs and liver, enlarged spleen and lymph nodes, cytokine production macrophages impaired, proliferative response B-cells increased.		[166, 167]
*PLEK*	*Plek*(-/-) mice	Platelets exhibit a defect in exocytosis, actin assembly and aggregation after PKC stimulation.		[168]
*PTGER4*	*Ptger4*(-/-) mice	Increased number of B-cells that have greater resistance to spontaneous cell death *in vitro* (accelerated B-cell tumor spread).		[169, 170]
*RGS1*	*Rgs1*(-/-) mice	B-cells respond excessively and desensitize improperly to chemokines CXCL12 and CXCL13.		[171]
*RPS6KB1*	p70s6k(-/-) mice	Smaller than wild-types and high levels of S6k1 in thymus.		[172, 173]
*SCO2*	KI/KO *Sco2* mice	Muscle weakness, respiratory chain deficiencies, complex IV assembly defects in multiple tissues and reduction in mitochondrial copper content.		[174]
*SOX8*	*Sox8*(-/-) mice	Weight reduction, however not attributable to significant structural deficits in any of the Sox8-expressing tissues.		[175]
*STAT3*	*Stat3*&amp;beta;(-/-) mice	Diminished recovery from endotoxic shock and hyperresponsiveness to endotoxin-inducible genes in liver.		[176, 177]
	Conditional* Stat3* knockout mice	Abnormalities in myeloid cells, overly pseudoactivated innate immune responses and Crohn's disease like pathogenesis.		[178]
	T-cell specific *Stat3*(-/-) mice	Deficient T-cells, severely impaired IL-6 induced proliferation.		[179]
*THEMIS*	*Themis*(-/-) mice	Impaired thymocytic positive and negative selection resulting in fewer mature thymocytes.		[180, 181]
	ENU mutant mice	Reduced SP thymocytes.		[182, 183]
*TNFRSF1A*	*p55* knock-in mice	Enhanced macrophage activation (spontaneous hepatitis and more susceptible to endotoxic shock, arthritis and EAE).	More susceptible to EAE after immunization.	[91]
	*p55*(-/-) mice	Less Th1 cytokines (IL-2, IFN-γ) but significant levels of IL-5 (Th2 phenotype).	Completely resistant against EAE.	[92, 93]
	*p75*(-/-) mice	Enhanced Th1 cytokine production (IL-2, IFN-γ) and enhanced CD4+ and F4/80+ central nervous system infiltration.	Exacerbated EAE.	[92, 93]
	*p55*(-/-) *p75*(-/-) mice		Become resistant to EAE.	[92]
*TNFSF14*	*Tnfsf14* KO = *Light*(-/-) mice	Defective IL-2 secretion of CD4+ T-cells and impaired proliferative responses of CD8+ T-cells but normal lytic effector function.&amp;nbsp;		[184-187]
*TYK2*	Natural missense mutation (B10.Q/J strain)	Defect in the generation of Th1 cells.	Become resistant to EAE.	[188-190]
	*Tyk2*(-/-) mice	Defective IL-12 induced T-cell function.	Become resistant to EAE.	[89, 191]
*VCAM1*	Endothelial and hematopoietic cell conditional* Vcam-1 *KO mice	Impaired lymphocyte migration to bone marrow.		[192]
*ZFP36L1*	*Zfp36l1*(-/-) *Zfp36l2*(-/-) mice	Perturbation of thymic development, higher expression of Notch1 in untransformed thymocytes, develop T-cell acute lymphoblastic leukemia.		[193]

Abbreviations: antibody (Ab), antigen (Ag), cyclic adenosine monophosphate (cAMP), double knockout (DKO), double positive (DP), experimental autoimmune encephalomyelitis (EAE), eN-ethyl N-nitrosourea (ENU), interferon (IFN), immunoglobulin (Ig), interleukin (IL), knock-in (KI), knockout (KO), single positive (SP), T cell receptor (TCR), tumor necrosis factor (TNF).

**Table 4.** Effects of MS Risk Alleles on Intermediate Phenotypes

Gene	Variant* Risk Allele (Location)	*In silico *Prediction	*In vitro *(Constructs, Cell Lines)	*Ex vivo *(Peripheral Blood Cells)
*CD40*	rs6074022* A (upstream)	Correlated with variant at -1 of translation start codon: disruption Kozak consensus sequence [65]		↓ mRNA expression in whole blood [194], ↓ protein expression [195]
*CD58 *	rs2300747* A (intronic)		↓ CD58 mRNA expression in lymphoblast cell lines [196]	↓ CD58 mRNA expression in PBMCs [196]
*CD6*	rs17824933* G (intronic)			↓ full-length expression in CD4+ and CD8+ T cells, ↓ CD4+ T cell proliferation [197]
HLA-DPB1* 0301	rs9277535* G (downstream)		↓ expression in lymphoblast cell lines [198]	↓ mRNA expression in liver [199]
*IL2RA (CD25)*	rs2104286* A (intronic)			↑ % of naive CD4+ T cells that are CD25+ and ↑ CD25 expression on stimulated CD14+CD16+ monocytes [98]
*IL7RA *	rs6897932* G (exonic)	Disrupts exonic splicing silencer [27]	↑ exon 6 splicing → ↑ soluble/transmembrane ratio [27]	↑ exon 6 splicing in PBMCs → ↑ soluble/transmembrane ratio [27]
*SP140*	rs10201872* A (intronic)		↓ expression in lymphoblast cell lines [200]	
*TNFRSF1A*	rs1800693* G (intronic)		↑ splicing of exon 6 [97]	↑ soluble receptor blocking TNF-α [97]
*TYK2 *	rs34536443* G (exonic)			↑ TYK2 phosphorylation in stimulated T cells, ↑ TYK2 activity, shift towards Th1 cytokine profile [90]

Abbreviation: peripheral blood mononuclear cells (PBMCs).
